# The Human Nucleolar Protein FTSJ3 Associates with NIP7 and Functions in Pre-rRNA Processing

**DOI:** 10.1371/journal.pone.0029174

**Published:** 2011-12-16

**Authors:** Luis G. Morello, Patricia P. Coltri, Alexandre J. C. Quaresma, Fernando M. Simabuco, Tereza C. L. Silva, Guramrit Singh, Jeffrey A. Nickerson, Carla C. Oliveira, Melissa J. Moore, Nilson I. T. Zanchin

**Affiliations:** 1 Laboratório Nacional de Biociências, Centro Nacional de Pesquisa em Energia e Materiais, Campinas, São Paulo, Brazil; 2 Department of Biochemistry, University of São Paulo, São Paulo, Brazil; 3 Department of Cell Biology and Cancer Center, University of Massachusetts Medical School, Worcester, Massachusetts, United States of America; 4 Department of Biochemistry and Molecular Pharmacology and Howard Hughes Medical Institute, University of Massachusetts Medical School, Worcester, Massachusetts, United States of America; 5 Instituto Carlos Chagas, Fundação Instituto Oswaldo Cruz, Curitiba, Paraná, Brazil; University of Edinburgh, United Kingdom

## Abstract

NIP7 is one of the many trans-acting factors required for eukaryotic ribosome biogenesis, which interacts with nascent pre-ribosomal particles and dissociates as they complete maturation and are exported to the cytoplasm. By using conditional knockdown, we have shown previously that yeast Nip7p is required primarily for 60S subunit synthesis while human NIP7 is involved in the biogenesis of 40S subunit. This raised the possibility that human NIP7 interacts with a different set of proteins as compared to the yeast protein. By using the yeast two-hybrid system we identified FTSJ3, a putative ortholog of yeast Spb1p, as a human NIP7-interacting protein. A functional association between NIP7 and FTSJ3 is further supported by colocalization and coimmunoprecipitation analyses. Conditional knockdown revealed that depletion of FTSJ3 affects cell proliferation and causes pre-rRNA processing defects. The major pre-rRNA processing defect involves accumulation of the 34S pre-rRNA encompassing from site A′ to site 2b. Accumulation of this pre-rRNA indicates that processing of sites A0, 1 and 2 are slower in cells depleted of FTSJ3 and implicates FTSJ3 in the pathway leading to 18S rRNA maturation as observed previously for NIP7. The results presented in this work indicate a close functional interaction between NIP7 and FTSJ3 during pre-rRNA processing and show that FTSJ3 participates in ribosome synthesis in human cells.

## Introduction

Synthesis of eukaryotic ribosomes takes place mainly in the nucleolus, a specialized cell compartment within the nucleus, where RNA polymerase I transcribes a large polycistronic ribosomal RNA, the 47S pre-rRNA. This pre-rRNA contains the 18S, 5.8S and 28S rRNAs flanked by the 5′ and 3′ external spacer sequences (5′ ETS and 3′ ETS) and by the internal spacer sequences 1 (ITS1) and 2 (ITS2). It is processed into the 18S, 5.8S and 28S mature rRNAs by a series of endo- and exonucleolytic cleavages and covalent nucleotide modifications concomitantly with assembling of ribosomal proteins to form the ribosomal particles. Pre-rRNA cleavages and modifications, which include base and ribose methylation and uridine isomerization to pseudouridine at specific sites, are mediated by trans-acting factors. These factors bind to nascent pre-ribosomal particles and dissociate as their function is accomplished along this high-energy consuming process [1]–[5]. Approximately 200 eukaryotic pre-ribosome trans-acting factors have already been identified based on protein interaction and genetic and biochemical analyses [5].

In *Saccharomyces cerevisiae*, mutations in genes required for ribosome biogenesis usually interfere with the order of pre-rRNA processing steps, causing accumulation of aberrant pre-rRNAs or fast degradation of pre-rRNA intermediates. Ribosome synthesis defects eventually lead to imbalance of the 40S/60S subunit ratio or affect subunit export to the cytoplasm. Ribosome synthesis and function have additional implications for multicellular organisms, especially for humans, where over fifteen genetic diseases have already been linked to mutations in genes that affect ribosome structure or synthesis [6]. These genes can be divided into three major groups. One group includes permanent components of ribosomes both from the small (RPS7, RPS14, RPS17, RPS19, RPS24) and large (RPL5, RPL11, RPL35A) subunits. A second group encodes trans-acting protein factors required for synthesis of both the small (UTP14c, CIRH1A, EMG1, WDR36, HCA66) and large (RBM28, SBDS) subunits. And, a third group includes components of small ribonucleoproteins involved in pre-rRNA cleavage (RMRP), pseudouridylation (DKC1, NOP10, NHP2) and methylation (TCOF1, HBII-85 – deleted Box C/D cluster) and in rDNA transcription (TCOF1) [7]. These genetic diseases underscore the importance of accurate ribosome synthesis and function for normal cellular function.

*S. cerevisiae* has been widely used as a model system to identify trans-acting factors and to study the ribosome synthesis mechanism. Although the general mechanism is conserved in all eukaryotes, several key differences between yeast and mammalians have emerged. In wild type *S.cerevisiae* strains, processing of the 35S pre-rRNA follows a 5′ to 3′ processing hierarchy where the 5′ ETS is cleaved before processing of ITS1, which in its turn is cleaved before ITS2 [2], [8], [9]. The mammalian 47S pre-rRNA, on the other hand, is initially converted to a 45S pre-rRNA that is processed by three simultaneous alternative pathways, depending on the site where the first cleavage occurs. In pathway A, the first cleavage at site 1 removes the complete 5′ ETS. In pathway B, the first cleavage takes place at site 2c in ITS1. In pathway C, the first site to be cleaved is 4b in the ITS2 [10], [11].

Most ribosome biogenesis factors have initially been characterized in *S. cerevisiae* and it has been widely assumed that their function is conserved in human cells. However, recent studies have shown that conditional depletion of human ribosome synthesis factors produce phenotypes significantly different from those observed in yeast. Bystin and hTsr1, the human orthologs of yeast Enp1 and Tsr1, respectively, are required for the maturation of the 18S rRNA and synthesis of the 40S subunit. However, conditional knockdown of these proteins in HEK293 lead to defects in pre-rRNA processing and 40S subunit export that are distinct from those reported for the yeast orthologs [12]–[15]. In *S. cerevisiae*, Nip7p depletion causes a profound effect on 60S subunit formation, leading to accumulation of unprocessed 27S pre-rRNA and to a deficit of 60S subunits [16]. Consistently with this, yeast Nip7p interacts with the Nop8p, Nop53p, Sdo1p proteins that are involved in 60S subunit synthesis [17]–[20]. Nip7p interacts also with Rrp43p, an exosome subunit involved in exonucleolytic maturation of the 3′-end of the 5.8S rRNA [18], [21]. In contrast to yeast, NIP7 knockdown in human cells leads to 40S ribosome deficiency. Pre-rRNA processing defects were detected in human cells depleted of NIP7, which include decrease of the 34S pre-rRNA and an increase of the 26S and 21S pre-rRNA concentrations [22].

The different phenotypes observed for conditional depletion of the yeast and human NIP7 proteins raised the possibility that the human NIP7 interacts with a different set of partners. To better understand the essential role played by NIP7 in ribosome biogenesis in human cells [22], and to elucidate the basis of functional difference displayed by NIP7 in yeast and humans, we performed a yeast two-hybrid screen to identify human proteins that interact with NIP7. We have identified FTSJ3 as a NIP7-interacting protein. FTSJ3 shows sequence similarity to the yeast protein Spb1. Both contain a putative RNA-methyl-transferase domain (FtsJ) in the N-terminal region and a conserved uncharacterized domain (Spb1_C) in the C-terminal region. Spb1 was shown to be required for 60S subunit synthesis in yeast [23] and to mediate methylation of the conserved G_2922_ that is located within the A loop of the catalytic center of the ribosome [24], [25]. A second line of evidence supporting the hypothesis that human NIP7 and FTSJ3 function in association during ribosome biogenesis as part of the same pre-ribosomal complexes comes from studies where both NIP7 and FTSJ3 were copurified by affinity purification of parvulin (Par14) [26]–[28].

The evidence mentioned above prompted us to further characterize the physical and functional interaction between NIP7 and FTSJ3. We describe in this study a functional association between NIP7 and FTSJ3 based on colocalization and coimmunoprecipitation analyses. We show also that FTSJ3 is required for pre-rRNA processing and cell proliferation, acting in the pathway leading to 18S rRNA maturation as observed previously for NIP7. Specifically, the cells depleted of FTSJ3 accumulate the 34S pre-rRNA, encompassing from site A′ to site 2b, indicating that processing of sites A0, 1 and 2 is inhibited in absence of FTSJ3.

## Results

### Isolation of FTSJ3 as a NIP7-interacting partner

**Figure 1:**
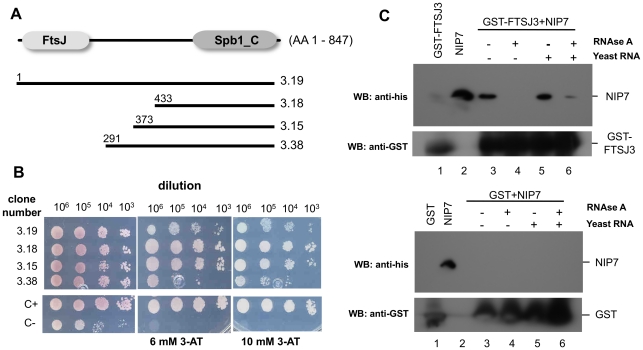
Isolation and validation of FTSJ3 as a bona-fide interaction partner of NIP7. (**A**) Diagram of the FTSJ3 protein (adapted from PFAM database) and cDNA clones showing positive interaction with NIP7 in the yeast two-hybrid system. Conserved domains, FtsJ and Spb1_C, are shown in grey boxes. Bars represent the length of each cDNA isolated in the yeast two-hybrid screen. The first amino acid encoded by the truncated cDNAs is indicated on the left. (**B**) Dilution-based growth assays of yeast cells tested for *HIS3* expression as a reporter of two-hybrid interactions between NIP7 and human cDNAs indicated on the left (top panel). A positive control for two-hybrid interaction (C+, yeast strain L40 expressing *lex*A-Nip7p and AD-Nop8p) and negative control (C−, yeast strain L40 expressing *lex*A-NIP7 and GAL4AD) are shown in the bottom panel. Dilutions of cells are shown on the top and 3-aminotriazole concentrations employed are indicated on the bottom. (**C**) Interaction assays using recombinant His-NIP7 and GST-FTSJ3. GST-FSTJ3 (upper panel) and GST (lower panel) were immobilized on gluthathione-sepharose beads and the indicated samples (+) were treated with RNase. Subsequently, purified His-NIP7 was added to the binding reactions. Yeast total RNA was also added to the indicated samples (+). Bound His-NIP7 was detected by immunoblotting using an anti-histidine antibody. Immunoblotting using a GST antibody was used to detect GST-FTSJ3 as indicated. RNase A treatment abolishes His-NIP7-GST-FTSJ3 interaction.

Human NIP7 plays an essential role in ribosome biogenesis and functions in close association with the SBDS protein [22], [29]. NIP7 knockdown in human cell lines leads to 40S ribosome deficiency with concomitant decrease of the 34S pre-rRNA concentration and an increase of the 26S and 21S pre-rRNA concentrations [22]. Increase of the 26S pre-rRNA indicates uncoupling of processing at sites A0 and 1 and increase of the 21S pre-rRNA indicates that processing at site 2 is particularly slower in NIP7-depleted cells. These defects are in contrast with those observed upon conditional depletion of Nip7p in yeast cells, which accumulate unprocessed 27S pre-rRNA and show a deficit of 60S subunits [16]. Given these observations, we hypothesized that the yeast and human NIP7 proteins display differential protein-protein interactions. To test such a hypothesis, we undertook a yeast two-hybrid screen to identify human NIP7-interacting proteins. The screen was performed using a l*ex*A-NIP7 fusion protein as bait to screen a human fetal brain cDNA library (Clontech HL4028AH). 121 positive clones were isolated from over 3×10^6^ yeast transformants. 50 positive clones were sequenced and ten of them (20%) encode FTSJ3, a putative ortholog of yeast Spb1p. FTSJ3 is an 847 amino acid protein containing a putative RNA-methyl-transferase domain (FtsJ, residues 22–202) and a Spb1_C domain (residues 640–847). The cDNAs isolated in the yeast-two hybrid screen encode both full-length and truncated proteins starting at residues 291, 373 and 433 (Fig. 1A). Expression of the two-hybrid reporters *HIS*3 (Fig. 1B) and *lac*Z (data not shown) confirmed interactions between NIP7 and FTSJ3 (full-length protein or its truncations) isolated in the screen. These results suggest that the FTSJ3 region comprising from residue 433 to the C-terminus mediates the interaction with NIP7. In order to confirm the interaction identified using the yeast two-hybrid system we performed pull-down assays using recombinant proteins produced in *E. coli*. The assay was performed with GST-FSTJ3 immobilized on glutathione-sepharose beads and purified histidine-tagged NIP7. As expected, NIP7 was retained in the bound fraction in the GST-FTSJ3 binding reaction (Fig. 1C, upper panel, lane 3) but not in the control reaction (Fig. 1C, lower panel, lane 3). The NIP7-FTSJ3 interaction observed both in the yeast two-hybrid and in the pull-down assay using recombinant proteins indicated that these proteins interact directly in vivo. However, we know from a previous study that NIP7 can bind to unspecific RNAs in vitro [22]. FTSJ3 possesses a putative RNA-methyl-transferase domain in the N-terminal region and a large intrinsically disordered region encompassing the central region and the conserved Spb1_C domain in the C-terminal region. These features indicate that FTSJ3 might also be an RNA-binding protein, raising the possibility that the interaction between NIP7 and FTSJ3 might as well take place through an RNA molecule. To test this possibility, a binding reaction was performed after treating the immobilized GST-FTSJ3 with RNase A. Interestingly, the interaction was abolished by the RNase A treatment (Fig. 1C, upper panel, lane 4). Addition of excess yeast total RNA to the binding reaction recovers NIP7 binding to GST-FTSJ3 (Fig. 1C, upper panel, lane 5). Treatment with RNase A after the addition of excess total RNA decreased the recovery of NIP7 interacting with FTSJ3 (Fig. 1C, upper panel, lane 6). These findings indicate that the interaction between recombinant NIP7 and FTSJ3 in vitro is mediated by binding to RNA molecules bound nonspecifically to both proteins.

### HEK293 endogenous NIP7 coprecipitates with FLAG-FTSJ3 through RNA

**Figure 2:**
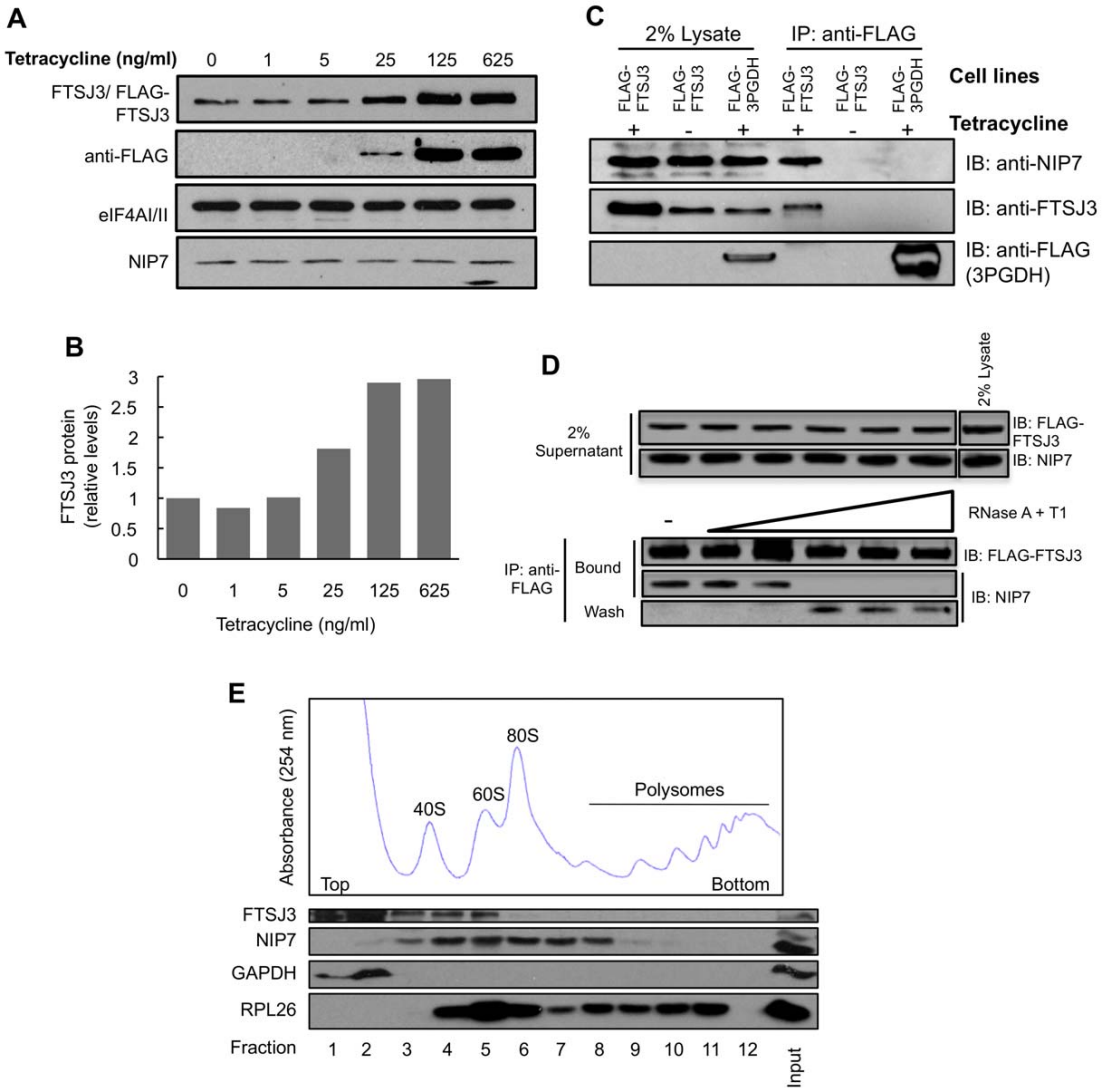
NIP7 associates with FTSJ3 in vivo in an RNA-dependent manner. (**A**) Analysis by western blot of FLAG-FTSJ3 induction in stably transfected HEK293 Flp-In T-Rex cells with increasing concentrations of tetracycline. Both endogenous and recombinant FLAG-FTSJ3 were detected by using antibody specific to FTSJ3. FLAG-FTSJ3 expression was confirmed by using antibody against the FLAG peptide. eIF4AI/II was used as a gel loading control. NIP7 levels are not affected by over-expression of FLAG-FTSJ3. (**B**) FTSJ3 relative levels as determined by band quantification using ImageJ software and normalized to eIF4AI/II. (**C**) Coimmunoprecipitation of NIP7 with FLAG-FTSJ3. FLAG-tagged FTSJ3 and 3PGDH were immunoprecipitated from extracts of stably transfected HEK293 Flp-In T-Rex cell lines using anti-FLAG (IP) followed by immunoblotting (IB) with anti-NIP7, anti-FTSJ3 and anti-FLAG. Parallel controls were performed using cells without induction and with cells stably transfected with FLAG-3PGDH. NIP7 is detected in the immunoprecitipation with FLAG-FTSJ3 (panel IP:anti-FLAG, lane + tetracycline) (**D**) FLAG-FTSJ3 was immunoprecipitated with anti-FLAG from cell extracts of stably transfected HEK293 Flp-In T-Rex treated with increasing concentrations of the RNases A and T1 and immunoblotted with antibodies for NIP7 and FTSJ3. RNase treatment abolishes NIP7 coimmunoprecipitation with FLAG-FTSJ3. (**E**) Analysis of FTSJ3 sedimentation. Cell extracts of HEK293 Flp-In T-Rex cells were fractionated by sucrose density gradient centrifugation and the fractions analyzed by immunoblotting using antibodies for the indicated proteins. FTSJ3 sedimentation overlaps with NIP7 in the range of the 40S–60S ribosome subunits. GAPDH was used as reference for soluble proteins and RPL26 as reference for 60S, 80S and polysome sedimentation.

The finding that the interaction between recombinant NIP7 and FTSJ3 is abolished by RNase treatment is intriguing and indicates that their interaction is also mediated by RNA in human cells. To test this possibility, we generated a stably transfected HEK293 derivative cell line that expresses N-terminally FLAG-tagged full-length FTSJ3 from a tetracycline-inducible promoter (Fig. 2A, B). A HEK293 cell line stably expressing an unrelated 3PGDH protein similarly fused to the FLAG-tag was used as a control. Following induction with tetracycline, we immunoprecipitated the FLAG-tagged proteins to test if endogenous NIP7 coimmunoprecipitated with FLAG-FTSJ3. NIP7 was detected in immunoprecipitates of FTSJ3 only when FLAG-FTSJ3 was induced with tetracycline prior to immunoprecipitation (IP) (Fig. 2C). Incubation of the extracts with RNase abolishes the coimmunoprecipitation of NIP7 with FLAG-FTSJ3, revealing that their biochemical association in HEK293 cells is dependent upon RNA (Fig. 2D). This finding strongly indicates that NIP7-FTSJ3 interaction in HEK293 cells does not take place by direct contact but is bridged by an RNA molecule. Alternatively, a molecular rearrangement caused by NIP7 binding to RNA is required for its interaction with FTSJ3.

Previous studies using sucrose density gradient fractionation showed that NIP7 cosediments with particles corresponding to pre-ribosomes [22]. Analysis of FTSJ3 sedimentation on sucrose density gradients revealed that a fraction of FTSJ3 overlaps with NIP7 in the range of the 40S–60S ribosome subunits although part of FTSJ3 is found in the soluble fractions (Fig. 2E). This result indicates that FTSJ3 and NIP7 are not components of a permanent complex but interact transiently with pre-ribosomal complexes during ribosome synthesis. This is consistent with the findings described below that FTSJ3 knockdown is required affects mainly the early processing steps while NIP7 is required for the late processing steps leading to 18S rRNA synthesis.

### FTSJ3 colocalizes with NIP7 in the nucleolus

**Figure 3:**
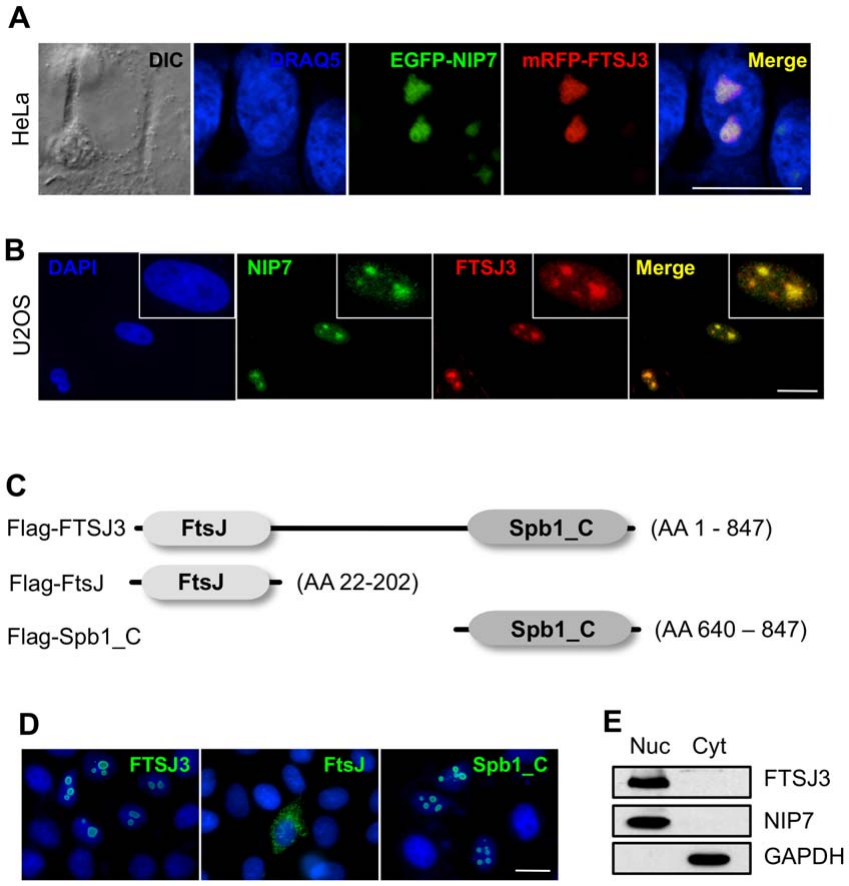
FTSJ3 colocalizes with NIP7 in the nucleolus. (**A**) Transient expression of EGFP-NIP7 and mRFP-FTSJ3 in HeLa cells. Both fusion proteins are observed in the nucleolus 48 hours post transfection. Nuclei were counterstained in blue with DRAQ5. (**B**) Immunofluorescent localization of endogenous NIP7 and FTSJ3 in U2OS cells. NIP7 and FTSJ3 were detected using secondary antibodies conjugated to Alexa Fluor 488 (green) and 568 (red), respectively. Nuclei were counterstained with DAPI. Boxes show an enlarged nucleus (**C**) Diagram of the FTSJ3 protein indicating the two conserved domains, FtsJ and Spb1_C (adapted from PFAM data base). (**D**) Subcellular localization of FLAG-tagged FTSJ3 and FtsJ and Spb1_C domains in transiently transfected HeLa cells. The FLAG-tagged proteins were immunostained with anti-FLAG followed by FITC-conjugated secondary IgG. Nuclei were counterstained with DAPI. The panel shows overlays of DAPI counterstaining and immunostaining. Scale bars represent 30 µm. (**E**) Western blots of HeLa nuclear (Nuc) and cytoplasmic (Cyt) preparations showing FTSJ3 in the nuclear fraction. NIP7 and GAPDH were used as controls for nuclear and cytoplasmic fractions, respectively.

To evaluate the potential functional association of NIP7 and FTSJ3 in vivo, we determined their subcelullar localization using exogenously expressed fluorescent fusion proteins and immunolocalization of the endogenous proteins. HeLa cells expressing NIP7 fused C-terminally to EGFP and RFP-tagged FTSJ3 were visualized by confocal microscopy for intrinsic fluorescence of the fusion proteins. Both exogenous proteins colocalized to the nucleolus (Fig. 3A). This colocalization of the two proteins to the nucleolus was independently confirmed by double staining of U2OS cells with antibodies against FTSJ3 and NIP7 (Fig. 3B).

The conserved domains found in FTSJ3 are represented in Fig. 3C. FTSJ3 contains a putative nuclear localization signal in its carboxy-terminal region (residues 808–847) that is part of the conserved Spb1_C domain. It is likely that this region of the protein is responsible for its localization to the nucleus, and perhaps the nucleolus. To test this, we assessed the subcellular localization of the Spb1_C domain (FTSJ3 residues 640–847) and FtsJ domain (residues 22–202), and compared those to the localization of the full-length protein. As seen in Figure 3D, cells transfected with FLAG-Spb1_C display nucleolar staining, similar to the cells that express FLAG-tagged full-length FTSJ3. In contrast, FLAG-FtsJ localized to the cytoplasm (Fig. 3D). These results suggest that Spb1_C domain mediates nucleolar localization of FTSJ3. Cell fractionation followed by immunoblot analysis showed that FTSJ3 is restricted to the nuclear compartment, which is consistent with its nucleolar localization (Fig. 3E). These findings further indicate a functional association between NIP7 and FTSJ3.

### FTSJ3 depletion affects NIP7 binding kinetics

**Figure 4:**
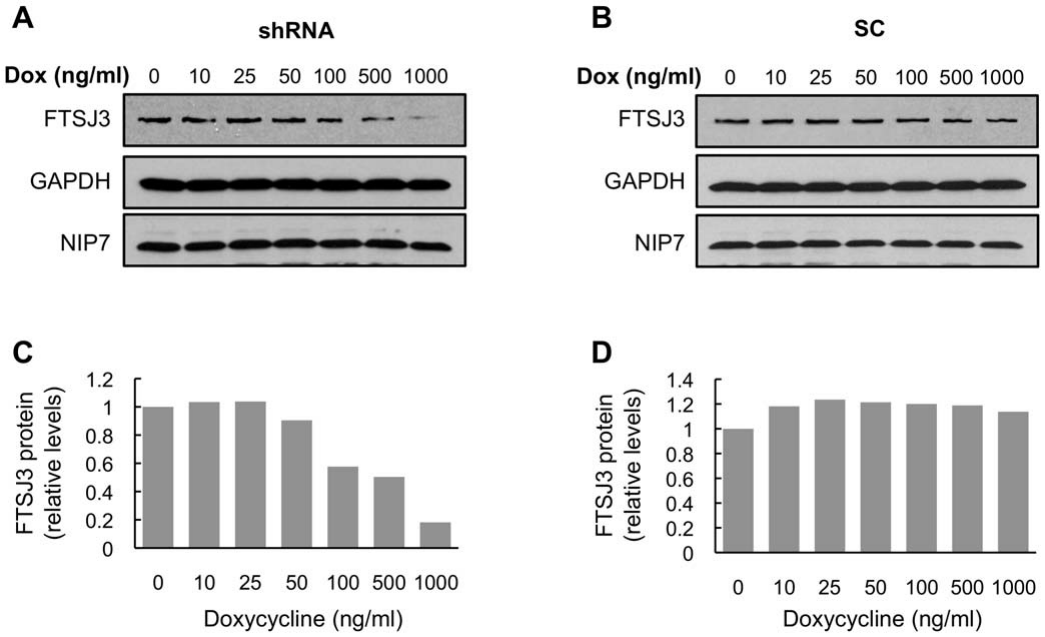
Analysis of FTSJ3 knockdown by RNA interference using the pFRT-U6tetO inducible system. (**A,** **B**) Western blot analysis to determine FTSJ3 levels in HEK293 Flp-In T-Rex cells expressing either a small hairpin RNAs targeting FTSJ3 (shRNA) or a scrambled control (SC) 72 h after induction with doxycycline (Dox). FTSJ3 levels decrease upon induction of the shRNA with doxycycline in dose-dependent manner. NIP7 levels are not affected by depletion of FTSJ3. (**C, D**) FTSJ3 relative levels were determined by band quantification using ImageJ software and normalized to GAPDH, which was used as an internal control. FTSJ3 was reduced to 20% of its original levels in cells treated with doxycycline at 1000 ng/ml.

**Figure 5:**
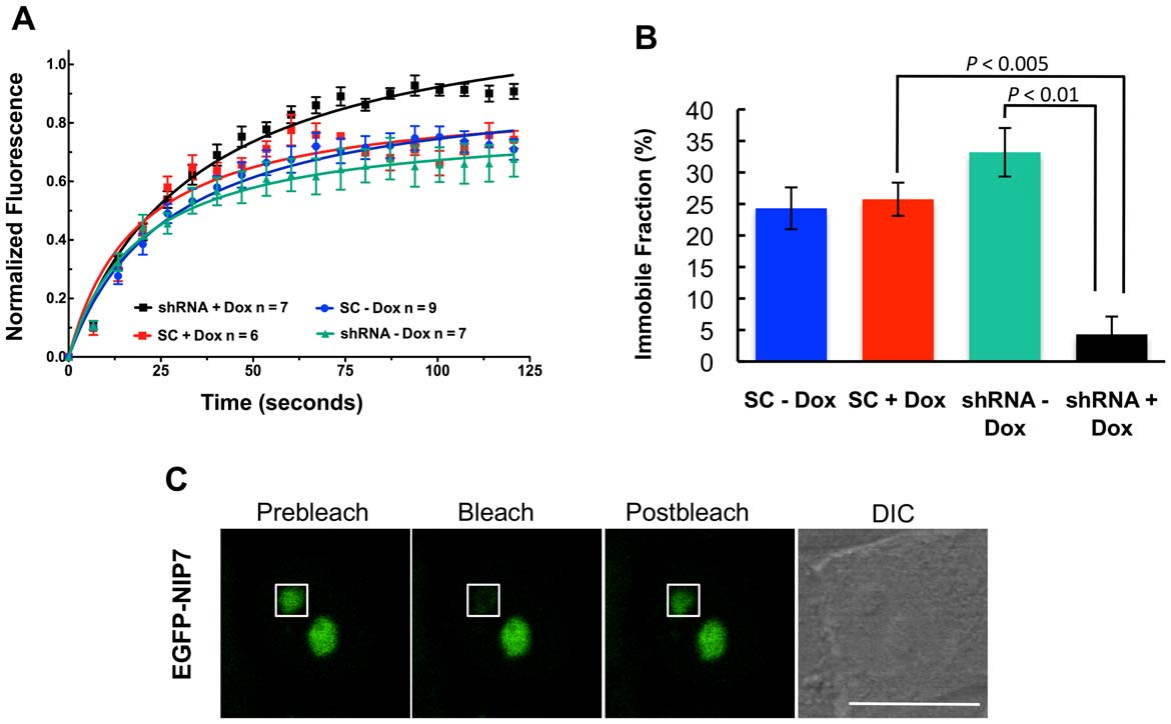
NIP7 shows different binding kinetics at the nucleolus under FTSJ3 depletion. (**A**) Fluorescence recovery after photobleaching (FRAP) was measured for EGFP-NIP7 in HEK293 Flp-In T-Rex cells expressing either a shRNA targeting FTSJ3 or a scrambled control (SC) and induced (+) or not (−) with doxycycline (Dox). The graph shows normalized mean fluorescence recovery curves over time calculated for EGFP-NIP7 at the nucleolus. Means were plotted with standard errors for each time point. (**B**) Graph of the 120-seconds time point postbleach. Data are representative of two independent experiments with at least 6 cells analyzed in each experiment. *P*-value was obtained by using a one-sided Student's t-test. (**C**) Images of a representative cell showing EGFP-NIP7 fluorescence before photobleaching (prebleach), immediately after photobleaching (bleach), and after 120 seconds when the fluorescence has already recovered (postbleach). The white box indicates the region of interest used in the analysis. The scale bar represents 30 µm.

If FTSJ3 and NIP7 function in close association in an RNP structure in the nucleolus, depletion of FTSJ3 might interfere with the kinetics of NIP7 binding to the nucleolar architecture. To address this question, we first generated stable HEK293 cell lines expressing a short-hairpin RNA (shRNA) targeting FTSJ3 mRNA, or scrambled shRNA as a control [30]. Western blots in Figure 4A and B (quantification in Fig. 4C and D) show that upon induction of the FTSJ3-targeting shRNA with doxycycline, the FTSJ3 protein levels are reduced to 18% of the control levels. Using these cells, we tested if there is a change in the immobile fraction of NIP7 in the nucleolus by measuring the fluorescence recovery after photobleaching (FRAP) of EGFP-NIP7 in the nucleolus. FRAP is used to measure the dynamics of molecular mobility, including diffusion, transport or movement of fluorescently labeled molecules. By subtracting the mobile fraction during a given time, it can also be used to determine the immobile labeled fraction. In this work, we used FRAP to determine the fraction of EGFP-NIP7 that is immobile in the nucleolus both in control and in FTSJ3 knockdown cells. As compared to the control (scrambled+Dox), the recovery of NIP7 fluorescence in the photobleached area (or nucleolus) was faster under FTSJ3 knockdown conditions (shRNA+Dox) (Fig. 5A). Quantification of fluorescent protein levels revealed that in the presence of FTSJ3, NIP7 was more tightly bound at the nucleolus, with the immobile fraction representing approximately 30% of the measured protein amounts (Fig. 5B). However, when FTSJ3 was depleted, the NIP7 dynamics changed, with a significant reduction in the protein's immobile fraction (∼4.3%, p<0.001) (Fig. 5B) probably caused. This analysis indicates that NIP7 binding to pre-ribosomes in the nucleolus is compromised in absence of FTSJ3 and is consistent with a close relationship of the proteins during ribosome biogenesis.

### Analysis of pre-rRNA processing intermediates in FTSJ3 knockdown cells

**Figure 6:**
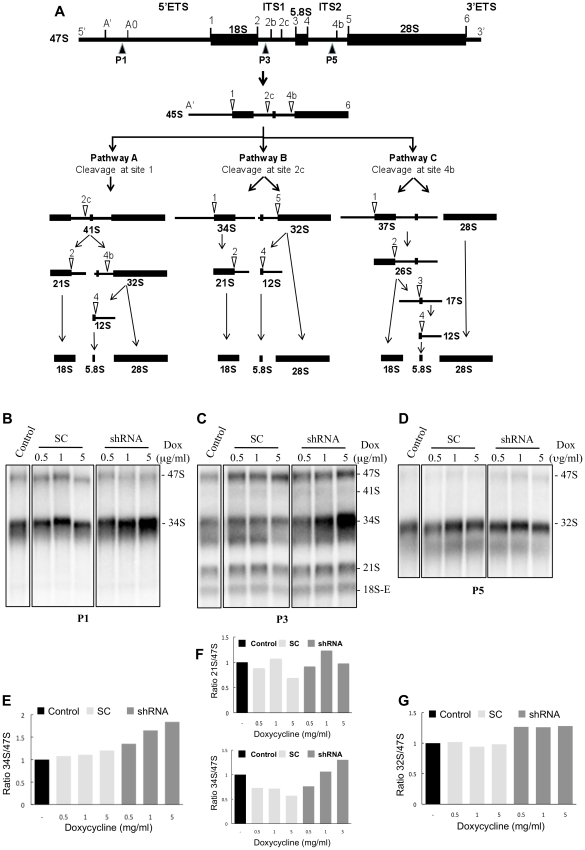
Analysis of pre-rRNAs intermediates in HEK293 Flp-In T-Rex cells expressing a shRNA targeting the FTSJ3 mRNA. (**A**) Diagram of the human pre-rRNA processing pathways. The 47S pre-rRNA is converted to the 45S pre-rRNA following the initial cleavages at sites A′ in the 5′-ETS and at site 6, at the 3′-end of the mature 28S rRNA. The 45S is processed by three alternative pathways, which is determined by the location of the first cleavage, leading to the mature rRNAs 18S, 5.8S and 28S. For additional details, see Morello et al. [22]. Open arrowheads indicate the sequential cleavage sites. Dark arrowheads (P1, P3 and P5) underneath the 47S pre-rRNA indicate the positions of the oligonucleotide probes used in Northern blotting. (**B**) Northern blot using probe P1 complementary to the 5′-ETS upstream site A0. (**C**) Northern blot using probe P3 complementary to ITS1 upstream site 2b. (**D**) Northern blot using probe P5 complementary to ITS2 upstream site 4b. (**E**–**G**) Graphs representing the ratio between the indicated intermediate pre-rRNA and the 47S pre-rRNA of the Northern blots shown in B (**E**), C (**F**) and D (**G**). Bands were quantified by using ImageQuant software. For Northern blots, RNA was isolated form HEK293 Flp-In T-Rex cells expressing either a shRNA targeting FTSJ3 (shRNA) or a scrambled control (SC) induced with the indicated concentrations of doxycycline (Dox) during 3 days. The control lane corresponds to the cell line expressing a shRNA targeting FTSJ3 not induced (−) with doxycycline.

Our observation of FTSJ3 interaction with NIP7, its nucleolar localization and sequence similarity to yeast Spb1 points to a role for FTSJ3 in pre-rRNA maturation in human cells. To investigate whether FTSJ3 is involved in pre-rRNA processing, we measured the steady-state levels of pre-rRNA intermediates in FTSJ3 knockdown cells. Initially, we analyzed pre-RNA processing using a HEK293 derivative cell line expressing a doxycycline-inducible shRNA that targets the FTSJ3 mRNA. Northern blots using total RNA extracted from these cells with probes complementary to the external and internal transcribed spacer sequences of the pre-rRNAs (Fig. 6) show a concomitant rise in steady-state levels of the 34S pre-rRNA in cells in which FTSJ3 knockdown was induced with increasing amounts of doxycycline for 3 days (Fig. 6B and C). A slight increase in the 21S pre-RNA levels can also be observed. Accumulation of the 34S pre-rRNA is detected by probes P1 and P3 showing that it encompasses from site A′ to site 2b which indicates that processing of sites A0, 1 and 2 are slower in cells depleted of FTSJ3. In comparison, no change in the levels of intermediates leading to 28S and 5.8S mature rRNAs was observed upon FTSJ3 knockdown (Fig. 6D). These findings indicate that FTSJ3 functions in the pathway leading to 18S rRNA maturation and 40S subunit synthesis and are consistent with a functional interaction with NIP7, which is also required for 18S pre-rRNA and 40S subunit formation [22].

**Figure 7:**
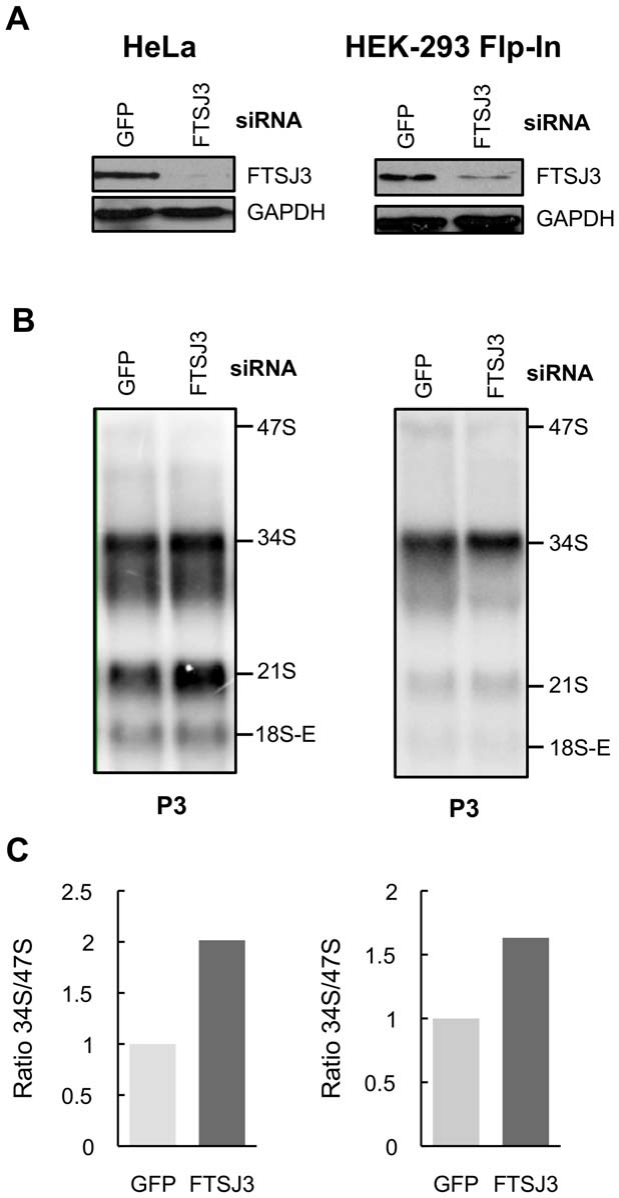
Analysis of pre-rRNAs intermediates in HeLa and HEK293 Flp-In T-Rex cells transiently transfected with a siRNA targeting the FTSJ3 mRNA. (**A**) Western blot analysis to determine FTSJ3 levels 72 h following siRNA transfection. (**B**) Northern blot using probe P3 complementary to ITS1 upstream site 2b. (**C**) Graphs representing the ratio between the 34S and 47S pre-rRNA of the Northern blots shown in B. Bands were quantified by using ImageQuant software. Control cells were transfected with siRNA targeting the GFP mRNA.

To confirm the results obtained with FTSJ3 knockdown using the doxycycline-inducible shRNA in HEK293 cells, we analyzed also the pre-RNA processing defects in HEK293 and HeLa cells transiently transfected with a siRNA directed to a different region of the FTSJ3 mRNA. FTSJ3 was efficiently knocked down in both cell lines (Fig. 7A). Northern blot analysis using probe 3, complementary to ITS1, revealed and increase of the 34S pre-RNA (Fig. 7B,C), which is consistent with the data obtained for the FTSJ3 knock down using the doxycycline-inducible shRNA.

### Conditional knockdown of FTSJ3 affects HEK293 cell proliferation rate

**Figure 8:**
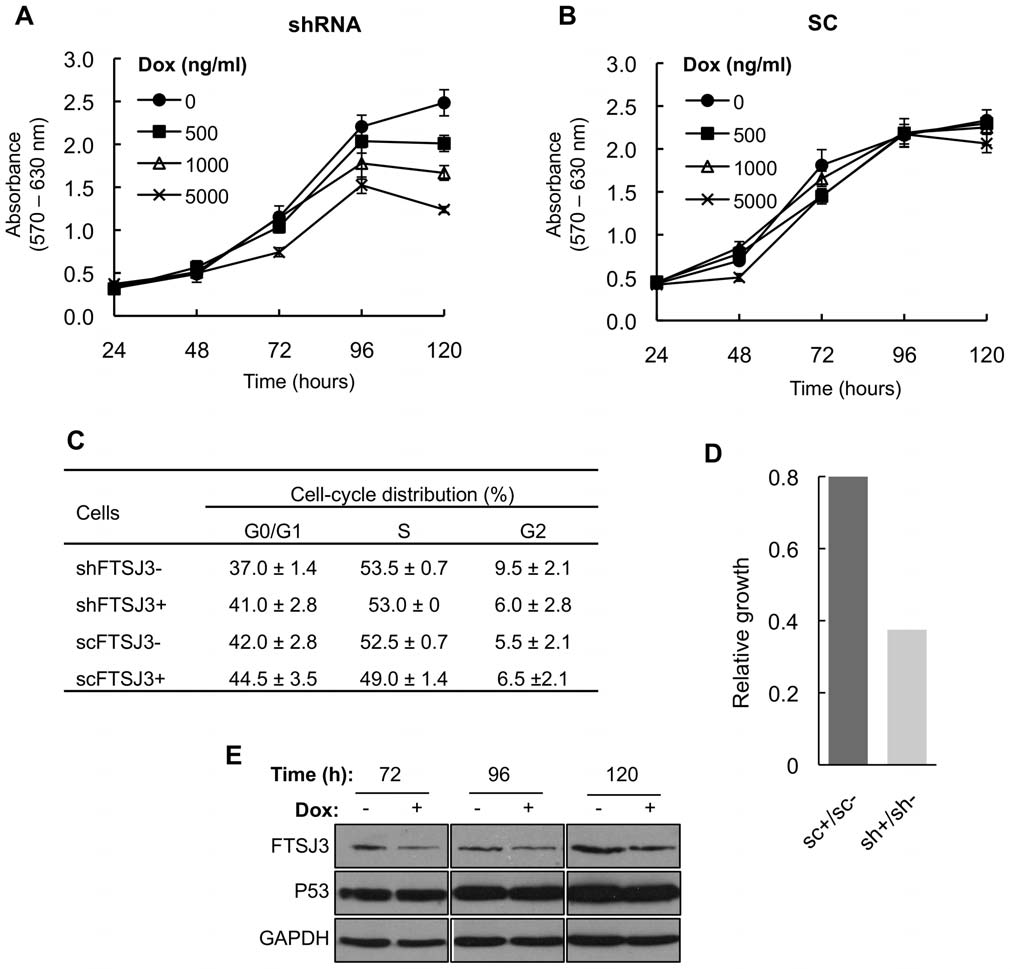
Analysis of cell proliferation over a 5-day period of induction with doxycycline (Dox). (**A**) Proliferation rate of cells expressing the shRNA targeting FTSJ3. (**B**) Proliferation rate of cells expressing the scrambled control SC RNA. (The graphs represent one of four independent assays performed using four technical replicates for each cell treatment). Induction of the shRNA targeting FTSJ3 leads to a reduction in cell proliferation. (**C**) Cell cycle analysis by fluorescence-activated cell sorting (FACS). FACS analyses were performed with HEK293 Flp-In T-Rex cells stably transfected with the pFRT-U6tetO plasmid expressing either a shRNA targeting FTSJ3 (shRNA) or a scrambled control (SC) and induced (+) or not (−) with 5 µg/ml of doxycycline for 5 days. (**D**) Relative growth rate differences between cells expressing the scrambled control scRNA (sc+/sc−) and cell expressing the shRNA targeting the FTSJ3 mRNA (sh+/sh−) induced (+) or not (−) with doxycycline for the 120 h time point. (**E**) Immunoblot analysis showing the levels of the FTSJ3 and p53 proteins in cells expressing either the shRNA targeting the FTSJ3 mRNA or the scrambled control induced (+) or not (−) with doxycycline. GAPDH was used as a gel loading control. The levels of FTSJ3 are reduced as expected and the levels of p53 are not affected.

Since downregulation of FTSJ3 affects NIP7 binding kinetics in the nucleolus and alters the balance of the pre-rRNA processing pathways causing an increase of the 34S pre-rRNA, we tested whether FTSJ3 knockdown affects cell proliferation. Cells expressing the FTSJ3-targeting shRNA showed a doxycycline dose-dependent reduction of the proliferation rate over 120 hours (Fig. 8A), whereas the proliferation of control cells expressing the scrambled RNA was not affected (Fig. 8B). This result indicates that prolonged depletion of FTSJ3 affects overall cellular physiology, reducing the proliferation rate. Nucleolar chemical stress and defective ribosome synthesis have been shown to trigger stress responses involving p53 which block cell cycle progression [31]–[35]. Fluorescence-activated cell sorting (FACS) analyses, however, did not reveal significant differences in the cell cycle stage of HEK293 cells expressing the shRNA targeting FTSJ3 as compared to the control cells expressing the scrambled control even though the cell proliferation rate of cells expressing the shRNA was reduced to nearly 50% of the proliferation rate of the control cells (Fig. 8C–E). The levels of p53 were similar in both test and control cells indicating that the reduced proliferation caused by FTSJ3 knockdown may not be related to activation of p53 and induction of apoptosis.

## Discussion

We have previously shown that the human NIP7 is involved in the pre-RNA processing pathway leading to 40S ribosomal subunit synthesis [22]. At the pre-rRNA processing level, human NIP7 knockdown leads to decrease of 34S pre-rRNA and increase of the 26S and 21S pre-rRNA concentrations. This imbalance in pre-rRNA concentrations is caused by uncoupling of processing at sites A0 and 1 and slower processing at site 2 in NIP7-depleted cells [22]. Despite of structure conservation, knockdown of human NIP7 affects 40S subunit formation while knockdown of yeast Nip7p leads to a deficit of 60S subunits showing that NIP7 acts at a different step of rRNA synthesis in these organisms [22], [36]. This finding raised the question whether the human and yeast orthologs share the same set of interacting partners. In this work, we have begun to test this hypothesis by employing the yeast two-hybrid system, which has widely been used to study protein interactions. Interestingly, FTSJ3 was isolated as one of the most frequent human NIP7-interacting candidate. This finding was intriguing because FTSJ3 is a putative ortholog of *S. cerevisiae* Spb1, which has previously been implicated in 60S subunit synthesis [23]. However, both studies describing protein composition of human pre-rRNA complexes [26], [27] along with the experimental evidence presented in this work on NIP7 and FTSJ3 interactions and their functional analysis are consistent with a major role for these proteins in the 40S biogenesis pathway in human cells. This is in contrast to the function of yeast Nip7p and Spb1 mainly in the pathway leading to 60S subunit synthesis.

In addition to the 60S synthesis defects caused by conditional depletion of yeast Nip7p, it has been found in pre-60S complexes [28], [37]–[39]. Similarly, Spb1 was shown to be required for 60S subunit synthesis in yeast [23] and to associate with pre-60S subunits [9], [28], [39]. So far, there is no report describing yeast Nip7p and Spb1 in pre-40S complexes [28], [40], [41]. Human NIP7 and FTSJ3, however, are found in complexes isolated by affinity purification of RPS19, a structural component of the mature 40S subunit [27]. Consistently with a function in 40S subunit synthesis, depletion of NIP7 and FTSJ3 was described to affect nuclear accumulation of RPS2-YFP, a small subunit reporter protein, but has little effect on the nuclear accumulation of a large subunit reporter protein, RPL29-GFP [42]. NIP7 and FTSJ3 are also found in association with parvulin and nucleolin complexes [26], [28], [43]. In these cases, association with a particular pre-ribosomal particle is less clear. Parvulin is present in both pre-40S and pre-60S complexes [26] while nucleolin has been proposed to function in the first step of pre-rRNA cleavage [44]. Complexes isolated by affinity purification of both parvulin and nucleolin contain trans-acting factors required for synthesis of both the 60S and 40S subunits [26], [28]. Association of NIP7 and FTSJ3 with parvulin and nucleolin complexes further supports their close functional association and might also suggest that at some point during assembly they both join pre-ribosomal complexes prior to separation of the pre-40S and pre-60S complexes.

The experimental data on subcellular localization and interaction analysis support a close functional relation between FTSJ3 and NIP7. Endogenous NIP7 and FTSJ3 and transiently transfected EGFP-NIP7 and mRFP-FTSJ3 fusion proteins are detected in the nucleolus (Fig. 3). In addition, FRAP analyses have shown that knockdown of FTSJ3 affects NIP7 binding dynamics in the nucleolus. We also showed FTSJ3 Spb1_C domain is responsible for nucle(ol)ar localization of FTSJ3. A GST pull-down assay performed with recombinant proteins indicated that they could interact directly. However, assays using bacterial expressed proteins and immunoprecipitation of FLAG-tagged FTSJ3 from HEK293 cell lysates showed that its interaction with NIP7 is mediated by RNA since incubation with RNase abolished the interaction in both cases (Figs. 1–2). NIP7 shows a conserved two-domain architecture and, for the *S. cerevisiae* and *P. abyssi* orthologs, the C-terminal PUA domain (after *P*seudo-*U*ridine synthases and *A*rchaeosine-specific transglycosylases) mediates RNA interaction [36]. The PUA domain is conserved in the human NIP7 and is likely responsible for the RNA-binding activity of NIP7 [22]. Structure predictions indicate that except for the putative RNA-methyl-transferase domain (residues 22–202) FTSJ3 contains a large intrinsically disordered region, roughly from amino acid 300 onwards until the C-terminus, which also includes the conserved uncharacterized domain (Spb1_C). We could not identify any RNA binding motifs in this region using different prediction algorithms, and the basis of FTSJ3's RNA binding remains an open question. Intrinsically disordered regions have higher flexibility and provide also larger binding interfaces when compared to folded proteins of the same size, allowing them to fit a variety of different binding partners [45]. The predicted structure of FTSJ3 is therefore compatible with NIP7 interaction mediated by an RNA molecule acting as a third partner.

The reduction in the proliferation rate of FTSJ3-knockdown cells shows that it plays an important cellular function (Fig. 8). Reduced proliferation arises most probably from defective pre-rRNA processing in absence of FTSJ3. FTSJ3 acts in the pathway leading to 18S formation (Figs. 6–7), the same pathway that requires NIP7 function [22]. However, our data argues for FTSJ3 function at distinct processing steps of this pathway as compared to NIP7. Accumulation of the 21S pre-rRNA is evidence for slower maturation of the 3′-end of the 18S rRNA (site 2) in NIP7-knockdown cells [22]. In mammalian cells, the cleavages of sites A0 and 1 in the 5′ ETS are coupled and defects that uncouple these cleavages lead to an increase of the 26S pre-rRNA, which is the case observed for NIP7 depletion [22]. Cells depleted of FTSJ3, on the other hand, accumulate unprocessed 34S pre-rRNA, which results from slower processing of the 5′ ETS sites A0, 1 and from slower processing of site 2 (Figs. 6–7). This finding indicates that FTSJ3 acts at earlier processing steps relative to NIP7. Recently, O'Donohue and co-workers [46] described the identification of two functional groups of 40S ribosomal proteins. One group, termed initiation-RPS, is required for the initial processing steps while the second group, termed progression-RPS is required for the late steps of 18S rRNA synthesis. Knockdown of individual members of the initiation-RPS group leads to a strong accumulation of the 34S pre-rRNAs, which results from inhibition of the 5′ ETS sites A0 and 1 and of the ITS1 site 2. On the other hand, knockdown of members of the progression-RPS group, show accumulation of the 26S, 21S and 18S-E pre-rRNAs [46]. A comparison of these defects with those caused by FTSJ3 and NIP7 depletion suggests that FTSJ3 acts during the stage of initiation-RPS while NIP7 acts during the stage of progression-RPS.

This work raised an intriguing question regarding the functional homology between *S. cerevisiae* Spb1 and human FTSJ3 which share 33% overall amino acid sequence identity and 52% similarity. Spb1 depletion causes decrease of the 27SA2 and 20S pre-rRNAs, appearance of a 23S aberrant pre-rRNA and accumulation of unprocessed 35S pre-rRNA [23]. In addition, Spb1 deficiency leads to formation of halfmer polysomes and deficit of 60S subunits [23]. Spb1 knockdown does not affect global rRNA methylation [23] but it is required for site-specific methylation at residue Gm_2922_ located in the catalytic center of the ribosome [24], [25]. Differences in specific pre-rRNA processing steps in *S. cerevisiae* and humans may be more common than initially thought. A recent study has shown that conditional knockdown of the human orthologs of yeast Enp1 and Tsr1, bystin and hTsr1 in HEK293 cells leads to defects in pre-rRNA processing and 40S subunit export that are distinct from those reported for the yeast orthologs [12]–[15]. Our findings regarding FTSJ3 and NIP7 function in 18S rRNA maturation in this and previous work add to the growing list of differences in yeast and human rRNA processing pathways [22]. Whereas our work on the role of NIP7 and FTSJ3 in pre-rRNA processing sheds new light on the mechanism of ribosome biogenesis in human cells, it also portends presence of other yet to be discovered differences in ribosome biogenesis pathways in yeast and mammals.

## Materials and Methods

### Plasmid construction and bacterial strains

Plasmids construction and cloning procedures are briefly summarized below. pTL1-NIP7 was generated by replacing the ampicilin *E. coli* selection marker of vector pBTM-NIP7 [17] to kanamycin. Plasmid pACT-NOP8 has been described previously [17]. The human NIP7 540 bp coding sequence was isolated from pTL1-HsNip7 [29] using the EcoRI/SalI restriction sites and inserted into the pET28a and pEGFP-C2 plasmids, generating pET28-HsNip7 and pEGFP-HsNip7, respectively. pACT-FTSJ3 was isolated in a yeast two-hybrid screen using the human NIP7 as bait (see below). The polylinker of pmRFP was modified to adjust the reading frame to that of the *FTSJ3* cDNA isolated from pACT-FTSJ3. pmRFP was modified by inserting a polylinker into the HindIII/SalI restriction sites, generating plasmid pmRFPL. The *FTSJ3* coding sequence was isolated from pACT-FTSJ3 using the EcoRI/XhoI restriction sites and inserted into pGEX-5X2 (GE Healthcare), generating plasmid pGEX-FTSJ3. Subsequently, the *FTSJ3* cDNA was isolated from pGEX-FTSJ3 and inserted into pmRFPL using the EcoRI/NotI restriction sites, generating pmRFPL-FTSJ3. The full-length FTSJ3 cDNA and its FtsJ (amino acids 22 to 202) and Spb1_C (amino acids 640 to 847) domains were cloned into pcDNA 3.1(+) containing a FLAG-tag upstream of the modified multiple cloning site (pcDNA-FLAG) using the EcoRI/XbaI restriction sites, generating the plasmids pcDNA-FLAG-FTSJ3, pcDNA-FLAG-FtsJ and pcDNA-FLAG-Spb1_C, respectively. FTSJ3 was also cloned into pcDNA5/FRT/TO containing a FLAG-tag upstream of the multiple cloning site (pcDNA5/FRT/TO-FLAG) using the HindIII/XhoI restriction sites, generating the plasmid pcDNA5/FRT/TO-FLAG-FTSJ3. *Escherichia coli* strains DH5α and BL21(DE3) were maintained in LB medium containing 50 mg/ml of the required antibiotic used in transformant selection and manipulated according to standard techniques [47]. The targets for *FTSJ3* small hairpin RNA (shRNA) were a 19-residue oligonucleotide whose sequence was selected based on the Dharmacon siDESIGN Center (Thermo Scientific/Dharmacon RNAi Technologies). It corresponds to nucleotides 993–1011 of the FTSJ3 mRNA target sequence (accession number NM_017647.2). The loop sequence (TTCAAGAGA) used to generate the hairpin was previously described by Ambion siRNA Target Finder. The oligonucleotide sequences to generate the shRNA against the *FTSJ3* mRNA correspond to LGM7 (5′ GAT CGC TAC TAA ACT GGA GAA CAA TTC AAG AGA TTG TTC TCC AGT TTA GTA GTT TTT TGT AC 3′) and LGM8 (5′ AAA AAA CTA CTA AAC TGG AGA ACA ATC TCT TGA ATT GTT CTC CAG TTT AGT AGC 3′) and the control-scrambled sequence (SC) corresponds to LGM9 (5′ GAT CGC AAT AGC TAG CTG AAA CAA TTC AAG AGA TTG TTT CAG CTA GCT ATT GTT TTT TGT AC 3′) and LGM10 (5′ AAA AAA CAA TAG CTA GCT GAA ACA ATC TCT TGA ATT GTT TCA GCT AGC TAT TGC 3′). The annealed oligonucleotides were cloned into pFRT-U6tetO [30] previously digested with BglII/KpnI, generating pFRT-U6tetO-shRNA-FTSJ3 and pFRT-U6tetO-SC-FTSJ3, respectively.

### Yeast two-hybrid assays

The yeast host strain L40 [*MAT*a *his3*D*200,trp1-901,leu2-3,311,ade2,lys2801am URA3*::(*lexAop*)*_8_-lacZ LYS2*::(*lexAop*)*_4_*-*HIS3*] [48] used in the two-hybrid analyses contains both yeast *HIS3* and *E. coli lac*Z genes as reporters for two-hybrid interaction integrated into the genome. An L40 derivative strain bearing plasmid pTL1-HsNip7, encoding a DNA-binding (DBD, *lexA*) fusion protein was transformed with a human fetal brain cDNA library constructed in the pACT2 vector (Clontech HL4028AH) using a PEG/lithium acetate mediated protocol (Matchmaker Yeast Protocol Handbook, Clontech). Transformants showing positive interaction were selected on YNB plates supplemented with adenine and 6 mM 3-AT (3-amino-triazol, Sigma). Subsequently, positive clones (His3^+^) were subjected to a second round of selection based on the activation of the reporter gene *lac*Z, using X-Gal filter assays. Plasmid pACT2 was rescued from the positive clones and the NIP7-interacting proteins identified by DNA sequencing followed by BLAST analyses (http://www.ncbi.nlm.nih.gov/BLAST/). The L40 strain expressing the yeast proteins Nip7p fused to the DNA binding domain of *lex*A (pTL1-NIP7) and Nop8p fused to activation domain of *GAL4* (pACT-NOP8) was used as a positive control in yeast two-hybrid assays [17]. The L40 strain bearing plasmid pTL1-HsNip7 and pACT2 was used as a negative control.

### Cell culture methods and shRNA-mediated knockdown

HEK293 Flp-In T-Rex (Invitrogen), HeLa (ATCC) and U2OS (ATCC) cells were maintained in high-glucose (4.5 g/l) Dulbecco's modified Eagle's medium supplemented with 2 mM glutamine, 10% fetal bovine serum and 100 U/ml penicillin and 100 µg/ml streptomycin. HEK293 Flp-In T-Rex cells were cultivated with tetracycline-free fetal bovine serum (GIBCO). The cells were cultured at 37°C in a humidified atmosphere with 5% CO_2_. Transfections were performed with Lipofectamine 2000 (Invitrogen), according to procedures described by the manufacturer. HEK293/Flp-In/FLAG-FTSJ3 and HEK293/Flp-In/FLAG-3PGDH are cell lines derivative of HEK293 Flp-In T-Rex (Invitrogen) that were generated as follows: HEK293 Flp-In T-Rex cells at 60% estimated confluency in 10-cm plates were cotransfected with pcDNA5/FRT/TO-FLAG-FTSJ3 (1 µg) and pOG44 (9 µg) encoding the Flp recombinase, generating the stably transfected HEK293/Flp-In/FLAG-FTSJ3 cell line. Two days after transfection, cells were diluted 1∶10 and 1∶5 in 10-cm plates and the incubation medium was supplemented with 100 µg/ml hygromycin B. The medium was replaced every two days. Resistant colonies were pooled and maintained in medium described above that was supplemented with 50 µg/ml hygromycin B. Transgene expression was induced by adding tetracycline at the indicated concentrations (0.001 to 0.625 µg/ml) to the cultures. The HEK293/Flp-In/FLAG-3PGDH cell line was generated in the same way. To generate polyclonal stable FTSJ3 knockdown cell lines, we stably transfected HEK293 Flp-In T-Rex cells with pFRT-U6tetO [30] that expressed shRNA targeting FTSJ3 mRNA (nucleotides 1568–1586), or scrambled shRNA as a control as described above. To verify depletion, FTSJ3 protein levels were assayed by immunoblotting as described below. Proliferation rates were determined by using the MTT-based CellTiter 96 Non-Radioactive Cell Proliferation Assay (Promega). Medium with inducer was replaced every two days for FTSJ3 knockdown experiments. For fluorescence assays, 1×10^5^ cells were seeded on sterile 18 mm coverslips, and for live cell experiments, 3×10^5^ cells were seeded on sterile 40 mm coverslips. Cell-cycle analyses were performed by fluorescence-activated cell sorting (FACS). For these assays, 0.5×10^6^ cells were fixed on ice in 70% (v/v) ethanol for 30 min, washed with PBS and the DNA content stained with 20 mg/ml propidium iodide in PBS in the presence of 1 mg/ml RNAse. Cells were analyzed on a FACSCalibur TM (BD Biosciences) equipped with a 488-nm argon laser. The fluorescence was measured through a 575/25 band pass filter. Cells doublets were removed using the FL2-Area and FL2-Width parameters. Data acquisition was performed using CellQuest software (BD Biosciences) and analysis using ModFit software (Verity Software). FACS analyses were performed with HEK293 Flp-In T-Rex cells stably transfected with the pFRT-U6tetO plasmid expressing either a shRNA targeting FTSJ3 (shRNA) or a scrambled control (SC) and induced (+) or not (−) with 5 µg/ml of doxycycline for 5 days and harvested at ∼50% confluency.

### Transient transfection

HEK293 Flp-In T-Rex and HeLa cells at 60% estimated confluency in 10-cm plates were transiently trasfected with 50 nM of siRNA duplexes against FTSJ3 by using DharmaFECT Duo Transfection Reagent (Dharmacon) according to the manufacture's instructions. The accession number of the FTSJ3 mRNA target sequence is NM_017647.2 The oligoribonucleotides used to generate the siRNAs duplexes were purchased from SIGMA (SASI_Hs01_00197929). Their sequences are 5′ GCC UUA UUG UGG GAG UGG A_d_T_d_T 3′ (oligo 1307693, sense) and 5′ UCC ACU CCC ACA AUA AGG C_d_T_d_T 3′ (oligo 1307694, anti-sense) and correspond to nucleotides 280 to 298 in the FTSJ3 target mRNA. Parallel control transfection assays were performed with 50 nM of siRNA duplexes against GFP (sense 5′ CUU GAC UUC AGC ACG CGU CUU 3′ and anti-sense 5′ GAC GCG UGC UGA AGU CAA GUU 3′). 72 hours after transfections, the cells were harvested and 1/10^th^ was used for protein analysis and 9/10^th^ processed for RNA analysis.

### Immunoblot analysis and antibodies

Proteins were resolved by SDS–PAGE and immunoblot was performed as described previously [22]. Primary antibodies and their dilutions to detect respective proteins were as follows: chicken polyclonal anti-FTSJ3 (Sigma) (1∶80,000), rabbit polyclonal anti-NIP7 (Abcam) (1∶4,000), rabbit polyclonal anti-eIF4AI/II (Santa Cruz Biotechnology) (1∶2,500), rabbit polyclonal anti-GAPDH (Abcam) (1∶8,000), rabbit polyclonal anti-RPL26 (1∶2,500) (Bethyl Laboratories), mouse monoclonal anti-FLAG M2 HRP-conjugated (Sigma) (1∶1,000), mouse monoclonal anti-polyhistidine (Sigma) (1∶5,000). Secondary antibodies were horseradish peroxidase-conjugated goat anti-mouse IgG (Thermo Scientific), goat anti-rabbit IgG (Thermo Scientific) and rabbit anti-chicken/turkey IgG (Sigma). The immunoblots were developed using the ECL western blotting analysis system (Thermo Scientific).

### Interaction assays using recombinant proteins

GST-FTSJ3 and His-NIP7 fusion proteins and GST used in control experiments were expressed in *E. coli* BL21(DE3) using the conditions described previously for His-NIP7 [22]. His-NIP7 was purified by metal-chelating chromatography as previously described [22]. GST-FTSJ3 and GST were isolated from IPTG-induced bacterial cell extracts as described below: bacterial cells were suspended in PBS buffer pH 7.5 (140 mM NaCl; 2.7 mM KCl; 10 mM Na_2_HPO_4_; 1.8 mM KH_2_PO_4_), containing 1 mM DTT, 1 mM PMSF, 0.5% (v/v) NP40 and 5% (v/v) glycerol. The cells were lysed by lysozyme treatment and sonication and extracts cleared by centrifugation. The extracts were incubated with glutathione-sepharose beads (GE Healthcare) for 1 hour at 4°C and the beads washed with 10 volumes of PBS buffer containing 1 mM DTT and 1 mM PMSF. Glutathione-sepharose beads containing either GST-FTSJ3 or GST were treated with RNAse A (100 U/ml) or with a mock-buffered solution for 30 min at 4°C. Subsequently the solution containing RNase A was removed and the beads washed once with 10 volumes of PBS buffer. 20 µg of His-NIP7 was added to the binding reactions and incubated for 2 h at 4°C. To the indicated samples, 5 µg of yeast total RNA were also added to the binding reactions. Subsequently the sepharose beads were washed with 10 volumes of PBS buffer with or without RNAse (as indicated) and the proteins analyzed by immunoblotting using mouse antibodies directed against the poly-histidine tag (Sigma) and rabbit antibodies to GST (Sigma).

### Cell fractionation

For isolation of nuclear and cytoplasmic extracts, 1×10^7^ cells were washed in ice-cold PBS and harvested by centrifugation at 500 *g* for 5 minutes at 4°C. Cells were lyzed in 1 ml of hypotonic cell lysis buffer [20 mM Tris–HCl, pH 7.5; 10 mM NaCl; 15 mM MgCl2; 1 mM EDTA; 0.5% (v/v) NP40; 0.1% (v/v) Triton X-100; complete EDTA-free protease inhibitor cocktail (Roche)] for 10 minutes on ice. Subsequently, NaCl was added to 150 mM final concentration and incubated on ice for 5 minutes. The lysate was centrifuged at 2,800 *g* for 10 minutes at 4°C, the supernatant was collected and cleared at 20,000 *g* for 10 minutes and the resulting supernatant was saved as the cytoplasmic extract. The pellet from the 2,800 *g* centrifugation, containing the nuclei, was washed once with hypotonic cell lysis buffer and the nuclear proteins extracted with 300 µl of denaturing buffer [hypotonic lysis buffer containing 1% (w/v) SDS]. The suspension was centrifuged at 20,000 *g* for 10 minutes at 4°C and the supernatant saved as the nuclear fraction.

### Co-immunoprecipitation assays

HEK293/Flp-In/FLAG-FTSJ3 and HEK293/Flp-In/FLAG-3PGDH cell lines were induced for 18–20 hours with 25 and 100 ng/ml of tetracycline, respectively. Cells from a 10-cm plate at 90% estimated confluency were suspended in 1 ml of hypotonic cell lysis buffer [20 mM Tris-HCl, pH 7.5; 15 mM NaCl; 10 mM EDTA; 0.5% (v/v) NP40; 0.5 mM DTT; complete EDTA-free protease inhibitor cocktail (Roche)] and incubated on ice for 10 minutes. NaCl was added to 150 mM final concentration and the lysate sonicated 10 times (2 second bursts) at 40% amplitude with at least 10 second intervals on ice. The cell lysate was cleared by centrifugation at 15,000 *g*. Subsequently, 50 µl of anti-FLAG M2 magnetic beads (Sigma) were added per ml of cell lysate and incubated in 1.5 ml centrifugation tubes on a rotating wheel for 2 hours at 4°C. The magnetic beads were harvested on a magnetic particle collector and supernatants were removed from the bead-bound material. The beads were washed 4 times with 1 ml of isotonic wash buffer (IsoWB) [20 mM Tris-HCl, pH 7.5; 150 mM NaCl; 0.5% (v/v) NP 40, complete EDTA-free protease inhibitor cocktail (Roche)] and suspended in 50 µl of IsoWB. For RNA-dependent protein interaction experiments, RNase A + T1 cocktail (AM2286 – Ambion) was titrated down (2.5 U RNase A and 100 U RNase T1, 0.25 U RNase A and 10 U RNase T1, 0.025 U RNase A and 1 U RNase T1, 0.0025 U RNase A and 0.1 U RNase T1 and 0.00025 U RNase A and 0.01 U RNase T1), and the bead suspension incubated in a water bath at 37°C for 10 minutes, and subsequently cooled for 5 minutes on ice. The supernatant was saved and beads were washed 3 times with 1 ml of IsoWB and suspended in 50 µl of SDS-PAGE loading buffer [47]. Proteins were detected by immunoblotting as described above.

### Immunocytochemistry and confocal microscopy

Immunofluorescent localization experiments to detect endogenous NIP7 and FTSJ3 were performed using U2OS cells as previously described [49]. Briefly, after washing in cold Hanks Balanced Salt Solution (HBSS) (GibcoBRL, Paisley, UK), cells were permeabilized in 0.5% Triton X-100 in CSK buffer (10 mM PIPES, pH 6.8; 300 mM sucrose; 100 mM NaCl; 3 mM MgCl_2_; 1 mM EGTA) for 5 minutes and subsequently fixed on ice with CSK buffer containing 4% formaldehyde for 50 minutes. U2OS cells were eventually stained with antibodies against NIP7 (Abcam) and FSTJ3 (Sigma), raised in rabbit and chicken, respectively, and with secondary goat anti-rabbit IgG conjugated with Alexa Fluor 488 (Invitrogen) and goat anti-chicken IgG conjugated with Alexa Fluor 568 (Invitrogen), both diluted 1∶100. Finally, the coverslips were analyzed on an inverted fluorescence microscope (Leica CTR6000). For immunolocalization of FLAG-tagged proteins, HeLa cells transfected with plasmids pcDNA-FLAG-FTSJ3, pcDNA-FLAG-FtsJ or pcDNA-FLAG-Spb1_C seeded on 18 mm coverslips were fixed in 4% paraformaldehyde in PBS, and permeabilized with 0.5% (v/v) Triton X-100 in PBS. Cells were subsequently incubated for 30 minutes in blocking buffer [0.2% (v/v) Triton X-100 and 3% (w/v) low fat milk in PBS], followed by incubation with mouse anti-FLAG M2 (Sigma) diluted 1∶100 in the same buffer for 1 hour at 37°C. Cells were washed three times with PBS containing 0.2% (v/v) Triton X-100 and incubated 1 hour at 37°C in blocking buffer with 1∶100 secondary anti-mouse coupled to FITC (Invitrogen). Finally, cells were washed as described above, counterstained in DAPI and mounted in PBS∶glycerol (1∶1). Images were obtained with a Nikon Eclipse E600 microscope and digital images were processed using the Cool-SNAPPro digital system (Media Cybernetics). For confocal microscopy, HeLa cells seeded on 18 mm coverslips were cotransfected with plasmids ecoding EGFP-NIP7 and mRFP-FTSJ3. 48 hours post transfection, cells were fixed with 4% paraformaldehyde for 20 minutes, permeabilized with 0.25% Triton X-100 in PBS for 10 minutes and mounted in ProLong Gold (Invitrogen) containing DRAQ5. The subcellular localization of EGFP- and mRFP- fusion proteins was monitored by fluorescence on a Leica confocal microscope (Leica Microsystems, Exton, PA).

### Fluorescence recovery after photobleaching (FRAP)

FRAP assays were performed at 37°C as previously described [49], [50]. Leica Confocal Software (Leica Microsystems, Exton, PA) was used to measure the intensity of fluorescence in the bleached region of interest and in the whole nucleus at each time point. Any remaining fluorescence in the bleached area after the bleach was normalized to zero. To calculate the relative fluorescence intensity (Irel) in the bleached area we used three alternative equations. The first one used the equation: Irel, t = N_0_*I_f_/N_t_*I_0_ [51]. The second approach used the equation: Irel, t = (I_t_*(N_0_/N_t_))−(I_pbl_*(N_0_/N_pbl_))/(I_0_−(I_pbl_*(N_0_/N_pbl_)). For both equations: N_0_ was the total nuclear fluorescence before bleaching, N_pbl_ was the total nuclear fluorescence in the first image taken after the bleach, N_t_ was the total nuclear fluorescence at time t, I_0_ was the fluorescence in the bleach zone before the bleach, I_t_ was the fluorescence in the bleach zone at time t, and I_pbl_ the fluorescence in the bleach zone in the first image taken after the bleach. Recovery curves were drawn using Graphpad Prism 5. Curve-fitting was performed as described previously [52]. Individual time points are presented as means with error bars showing standard errors. Several equations were tested, but the best fit for these photobleach recoveries was obtained using an exponential association curve: F(t) = Fmax (1−e^−kt^). All half times of recovery and immobile fractions were calculated from a best fit to this equation.

### Polysome profile analysis

Polysome profiles were analyzed on sucrose gradients essentially as previously described [22], [53]. Briefly, HEK293 Flp-In T-Rex cells stably transfected with the pFRT-U6tetO plasmid expressing either a shRNA targeting FTSJ3 (shRNA) or a scrambled control (SC) were induced (+) or not (−) with 5 µg/ml of doxycycline for 3 days and harvested at ∼50% confluency. Following addition of 100 µg/ml cycloheximide, 5×10^7^ cells were collected and lyzed using 500 µl of polysome buffer (PB) containing 20 mM Tris-HCl pH, 7.5; 100 mM NaCl; 10 mM MgCl_2_; 1 mM DTT; 1% (v/v) Triton X-100 and 100 µg/ml cycloheximide. Extracts were clarified by centrifugation at 20,000 *g* for 10 minutes at 4°C. Totals of 35 OD_260_ units were loaded onto linear sucrose gradients (10–50%) prepared in PB. Polysomes were separated by centrifugation at 40,000 rpm for 4 hours at 4°C using a Beckman SW41 rotor. Gradients were fractionated by monitoring absorbance at 254 nm. Protein precipitation and removal of sucrose for immunoblot analyses was performed as follows: 150 µl of each sucrose gradient fraction were mixed with 600 µl of methanol and subsequently mixed with 150 µl of chloroform; 450 µl of water were added to the mix and centrifuged at 20,000 *g* for 5 min at 4°C. The aqueous layer was discarded and the pellet washed with 650 µL of methanol, followed by centrifugation as described above. The liquid was discarded and the pellet was taken up in protein sample buffer and analyzed by immunoblotting.

### RNA analysis

Total RNA from HEK293/Flp-In/shRNA-FTSJ3 and HEK293/Flp-In/SC-FTSJ3 cell lines and from HeLa and HEK293 Flp-In T-Rex cells transiently transfected with siRNAs was isolated by TRI Reagent RT (Molecular Research Center, Inc.). RNA samples were fractionated by electrophoresis on 1.0% (w/v) agarose/formaldehyde gels, followed by transfer to Biotrans(+) nylon membranes (MP Biomedicals). Membranes were hybridized with [^32^P]-labeled oligonucleotide probes P1 (5′ CCC CAA GGC ACG CCT CTC AGA TCG CTA GAG AAG GCT TTT C 3′), P3 (5′ AAG GGG TCT TTA AAC CTC CGC GCC GGA ACG CGC TAG GTA C 3′) and P5 (5′ CGG GAA CTC GGC CCG AGC CGG CTC TCT CTT TCC CTC TCC G 3′) as described previously (Morello et al., 2011), for 16 hours in ExpressHyb solution (BD Biosciences). Northern blots were visualized and quantified using a Storm 840 Phosphorimager (Molecular Dynamics).
